# Bowel ultrasound scan in the management of pediatric moderate ulcerative colitis: The applicability of international bowel ultrasound segmental activity score

**DOI:** 10.1002/jpn3.70476

**Published:** 2026-06-08

**Authors:** Martina Di Benedetto, Luca Scarallo, Camilla Pannacci, Sara Renzo, Monica Paci, Sara Naldini, Jacopo Barp, Laura Tasciotti, Paolo Lionetti

**Affiliations:** ^1^ Gastroenterology and Nutrition Unit Meyer Children′s Hospital IRCCS Florence Italy; ^2^ Department of NEUROFARBA University of Florence Florence Italy; ^3^ Radiology Unit Meyer Children′s Hospital IRCCS Florence Italy

**Keywords:** IBD imaging, non‐invasive monitoring, pediatric IBD, risk stratification

## Abstract

**Objectives:**

To evaluate bowel ultrasound (BUS) findings and the diagnostic and prognostic performance of international bowel ultrasound segmental activity score (IBUS‐SAS) in children with moderate ulcerative colitis (UC), including its accuracy for assessing endoscopic activity and predicting clinical remission and colectomy within 12 months.

**Methods:**

This was a single‐center retrospective study conducted at Meyer Children′s Hospital IRCCS. All patients who underwent a BUS performed for moderate UC (defined as pediatric ulcerative colitis activity index 35–60, either at disease onset or at relapse) between January 2019 and December 2023 were included. Where available, data on ileocolonoscopy performed within 4 weeks before or after BUS were retrieved, if no changes in medical management were performed during this period.

**Results:**

Seventy‐seven patients were included; 35/77 (45.5%) underwent ileocolonoscopy within 4 weeks from BUS. The most common BUS finding was increased colonic wall thickness (74/77, 96.1%), followed by enhanced vascularization (70/77, 90.9%). Loss of wall stratification was observed in 15 of 77 BUS (19.5%). The discriminative ability of IBUS‐SAS in identifying moderate endoscopic activity was appraised through a receiver operating characteristic curve, showing a good area under the curve (0.75, 95% confidence interval [CI]: 0.58–0.90, *p* = 0.021) with an optimal cut‐off of 48.5. IBUS‐SAS ≥ 49 (as nominal variable) was identified as independent predictor of clinical remission (adjusted hazard ratio:0.42, 95% CI: 0.23–0.78) and colectomy within 12 months (adjusted odds ratio:4.18, 95% CI: 1.03–16.91).

**Conclusions:**

BUS is a non‐invasive, repeatable tool for monitoring disease activity and stratifying pediatric UC with moderate activity. IBUS‐SAS showed good discriminative ability for endoscopic activity, supporting its use for ultrasound‐based risk‐stratification in this setting.

## Abstract (highlights)

**What is Known:**

- Bowel ultrasound (BUS) has emerged as a significant non‐invasive tool for monitoring inflammatory bowel disease (IBD) in children.
- Current guidelines do not provide specific recommendations on how to integrate BUS into the management of patients with moderate disease activity.

**What is New:**

- This study validates the use of the international bowel ultrasound segmental activity score (IBUS‐SAS) score in children with moderately active ulcerative colitis (UC), demonstrating its ability to predict both clinical remission and colectomy within 12 months.
- Incorporating BUS and the IBUS‐SAS score into routine monitoring may enhance early risk stratification and support personalized treatment strategies in pediatric patients with moderate UC.

## 1 INTRODUCTION

The most recent European Society for Pediatric Gastroenterology, Hepatology, and Nutrition (ESPGHAN) guidelines recommend the pediatric ulcerative colitis activity index (PUCAI), fecal calprotectin, and endoscopy as the primary tools for assessing disease activity in pediatric ulcerative colitis (UC).^1^, ^2^ However, pediatric patients classified as having moderate UC according to PUCAI values (35–60) represent a prognostically heterogeneous group, with markedly different disease courses and therapeutic needs.^3^, ^4^, ^5^, ^6^ Identifying additional tools to further stratify these patients is therefore clinically important.

In recent years, bowel ultrasound (BUS) has emerged as a significant non‐invasive tool for monitoring inflammatory bowel disease (IBD) in both adults and children, due to its high patient tolerability and elevated diagnostic accuracy.^7^, ^8^, ^9^ Initially, its application in UC was limited, as UC was traditionally considered a predominantly mucosal disease affecting the distal colon.^10^ Nevertheless, recent advancements have led to an increasing recognition of the utility of BUS in UC, thanks to its strong correlation with endoscopic activity, its ability to effectively grade severity of inflammation, and its capacity to monitor response to treatments.^11^, ^12^, ^13^, ^14^, ^15^, ^16^

Given the heterogeneity of patients with clinical moderate disease activity, composite ultrasound scores, rather than isolated sonographic parameters, may be more effective in capturing subtle differences in disease activity and improving risk stratification. Several ultrasound‐based indices have been proposed for assessing disease severity in UC for adults,^12^, ^17^, ^18^, ^19^ including the Milan ultrasound score (MUC)^17^ and the UC‐intestinal ultrasound (IUS) index.^18^ However, the only score specifically developed for pediatric UC patients is the Civitelli index,^20^ a primarily qualitative tool, that may be insensitive to subtle but clinically relevant changes in disease activity due to his final score ranging from 0 to 4. In this context, the international bowel ultrasound segmental activity score (IBUS‐SAS),^21^ initially validated in Crohn′s disease (CD), has recently been evaluated in adult UC cohorts, showing a good correlation with endoscopic activity^22^, ^23^ Owing to its granular 0–100 scoring, IBUS‐SAS provides a quantitative framework that may allow more sensitive detection of changes over time.

Building on our prior experience with BUS in acute severe colitis (ASC),^15^, ^24^ we designed this study to characterize BUS findings and to determine the diagnostic and prognostic performance of the IBUS‐SAS in children with clinically moderate UC, using endoscopy as the reference standard and clinical outcomes as measures of prognostic validity.

## 2 METHODS

### 2.1 Ethics statement

The study protocol conforms to the ethical guidelines of the 1975 Declaration of Helsinki (6th revision, 2008). Ethical approval was obtained by Meyer Children′s Hospital IRCCS IRB (*approval protocol number: 180/2020*). Informed consent was obtained for every patient included in the study.

### 2.2 Population and setting

This single‐center retrospective study included all children followed up at our tertiary referral center (Meyer Children′s Hospital IRCCS, Florence, Italy) with moderate UC (PUCAI 35–60) either at diagnosis or at disease relapse, between January 1, 2019, and December 31, 2023, who met the following inclusion criteria: (i) having a BUS performed while on moderate disease activity (ii) a minimum follow‐up of 12 months. For patients enrolled during disease relapse, an additional inclusion criterion was applied, allowing only those experiencing relapse despite ongoing stable medical therapy, defined as treatment with corticosteroids or biologics for at least 1 month, or immunomodulators for at least 3 months.

The diagnosis of UC in all patients was confirmed through colonoscopy and biopsy, following established clinical, endoscopic, and histological criteria.^1^, ^25^ The following clinical, demographic, and laboratory data were extracted from each patient′s medical record at diagnosis, at the BUS time, and at the last available follow‐up: birth date, sex, age, C‐reactive protein (PCR), fecal calprotectin, PUCAI, and current stable therapy. Disease classification and behavior were defined according to the Paris classification.^26^

BUS is routinely performed at our hospital as part of the diagnostic workup for UC. Ultrasounds were all performed using a Philips IU22 machine with convex (5 MHz) and linear (7.5 MHz) probes and by a single pediatric radiologist with more than 20 years′ experience in performing BUS in IBD (L.T.). All ultrasound examinations in our center are performed using a structured reporting system that prospectively includes: colonic wall thickness (CWT), loss of colonic wall stratification (CWS), color Doppler signal (CDS), presence of enlarged mesenteric lymph nodes (short axis >5 mm), and inflammatory mesenteric fat (i‐fat). CWT was measured from the outer wall to the inner echogenic lumen or central mucosal stripe; two longitudinal and two transverse measurements were obtained for each segment, and mean values were calculated. Colonic wall vascularization was assessed using color and power Doppler and graded according to the Limberg score (0–4).^27^ Inflammatory mesenteric fat was defined qualitatively (present/absent) as the presence of hyperechoic, non‐compressible pericolic fat adjacent to an inflamed bowel segment, exceeding the expected physiological pericolic fat and associated with inflammatory changes, for each colonic segment. All ultrasound parameters were recorded for the ascending, transverse, descending, and sigmoid colon and the worst value across segments was used for analysis. Data were extracted from ultrasound examination reports; when clarification was needed, stored images were retrospectively reviewed together with the performing radiologist to confirm the reported findings, and no missing data were present for the ultrasound parameters analyzed.

The IBUS‐SAS was retrospectively calculated by two independent reviewers (M.D.B. and C.P.) through shared consensus,^21^ using routinely and systematically documented ultrasound parameters. The IBUS‐SAS includes four parameters: CWT, i‐fat, CDS, and CWS. The score was calculated using the following formula: IBUS‐SAS (0–100) = 4 · CWT + 15 · i‐fat + 7 · CDS + 4 · CWS. It was calculated for the colonic segment showing the most severe abnormalities.

To compare IBUS‐SAS with other ultrasound indices, we evaluated disease‐specific scores. However, only the MUC^17^ could be retrospectively derived from routinely recorded parameters. The Civitelli index^20^ and UC‐IUS^18^ were not applicable, as they require assessment of haustration, which is not routinely documented in our reports.

Where available, data on ileocolonoscopy performed within 4 weeks before or after the ultrasound were retrieved, if no change in medical management was performed during this period. Endoscopic activity was assessed using both the Mayo endoscopic subscore (MES) and the ulcerative colitis endoscopic index of severity (UCEIS),^28^, ^29^ as per standard of care in our institution.

### 2.3 Study outcomes

This study aimed to characterize BUS findings in children with clinically moderate UC and to determine the diagnostic accuracy of the IBUS‐SAS for assessing disease activity, using endoscopic evaluation as the reference standard. Additionally, we investigated the prognostic value of IBUS‐SAS for subsequent clinical remission (defined as PUCAI < 10) and the risk of colectomy within 12 months following BUS.

### 2.4 Statistical analysis

Data were managed and analyzed using IBM SPSS Statistics (version 25th). Categorical variables are reported as frequencies and percentages, and continuous variables as medians with interquartile ranges (IQR), as none followed a normal distribution (Kolmogorov–Smirnov test). Comparisons between categorical variables were performed using the *χ* ^2^ or Fisher′s exact test, as appropriate, while continuous variables were compared using the Mann–Whitney *U* test. For the subgroup of patients who had a BUS and ileocolonoscopy within 4 weeks, the correlation between IBUS‐SAS and UCEIS was assessed using Spearman′s rank correlation coefficient (*ρ*). The diagnostic accuracy of IBUS‐SAS, MUC, and CWT for identifying moderate endoscopic activity (UCEIS ≥ 5) was assessed using ROC curve analysis. The optimal IBUS‐SAS cut‐off was determined using the Youden Index (J). Based on this cut‐off, patients were stratified into two groups (IBUS‐SAS below vs. above cut‐off), and clinical, laboratory, and outcome variables were compared, including disease characteristics, treatment, need for therapeutic escalation, hospitalization, clinical remission, and colectomy within 12 months. Kaplan–Meier analysis was used to estimate clinical remission and colectomy‐free survival, with comparisons by log‐rank test. Predictors of clinical remission and colectomy within 12 months following the BUS assessment were assessed using Cox regression (expressed as hazard ratio [HR] with 95% confidence intervals [CI]) and logistic regression (expressed as odds ratio [OR] with 95% CI) univariable and multivariable models, respectively. All statistical tests were two‐sided and a *p*‐value < 0.05 was considered as the statistically significant threshold. No formal a priori sample size calculation was performed for this analysis, as this was a retrospective study including all consecutive eligible patients during the study period. The reporting of this study conforms to the Strengthening the Reporting of Observational Studies in Epidemiology (STROBE) statement.

## 3 RESULTS

A total of 77 patients (37 [48.1%] males; median age at inclusion, 12 years [IQR 9.2–13.8]) were included. Clinical data, BUS findings and endoscopic features at BUS time are summarized in Table 1. The median PUCAI at the time of the ultrasound examination was 45 (IQR 40–55). At BUS time, 25 patients (32.5%) were receiving first‐line anti–tumor necrosis factor alpha therapy and 8 (10.4%) second‐line biologics (vedolizumab or ustekinumab). A total of 14 patients (18.2%) had previously experienced a severe disease flare. Overall, 35 (45.5%) BUS were performed at disease onset and 42 (54.5%) during relapse.

**Table 1:** Clinical characteristics, BUS findings, and endoscopic features at BUS time.

Characteristics	Overall cohort (*n* = 77)
Median PUCAI at BUS time	45 (IQR: 40–55)
Median CRP at BUS time (mg/dL)	1.03 (IQR: 0.6–3.2)
Median fecal calprotectin at BUS time (µg/g)	650 (IQR: 470–1500)
Paris classification at BUS time	
Disease′s extension (E)	
E1	1 (1.3%)
E2	18 (23.4%)
E3	7 (9.1%)
E4	51 (66.2%)
Severe acute relapse (S)	
S0	63 (81.8%)
S1	14 (18.2%)
Concomitant stable therapy at BUS	
No medication	25 (32.5%)
Steroids	35 (45.5%)
Mesalamine	47 (61%)
Azathioprine	15 (19.5%)
Anti‐TNF‐α agents	25 (32.5%)
Infliximab	22 (28.6%)
Adalimumab	3 (3.9%)
No anti‐TNF‐α agents	8 (10.4%)
Vedolizumab	7 (9.1%)
Ustekinumab	1 (1.3%)
BUS indication	
Disease′s onset	35 (45.5%)
Disease′s Relapse	42 (54.5%)
Median time between diagnosis and BUS (months)	2.9 (0.1–14.8)
BUS findings (77/77)	
Thickened colonic wall (present)	74 (96.1%)
Median colonic wall thickness (mm)	5 (IQR: 4‐6.5)
Enhanced vascularization (present)	70 (90.9%)
Colonic wall stratification loss (present)	15 (19.5%)
Inflammatory mesenteric fat (present)	42 (54.5%)
Enlarged mesenteric lymph nodes (present)	51 (66.2%)
Median Limberg score	3 (IQR: 2.5–3)
Median MUC	8.3 (IQR: 7.6–11.2)
Median IBUS‐SAS score	52 (IQR: 34–70)
Mayo endoscopic score ± 4 weeks to BUS (35/77)	
Median value	2 (IQR: 2–3)
1 (mild)	6 (17.1%)
2 (moderate)	14 (40%)
3 (severe)	15 (42.9%)
UCEIS ± 4 weeks to BUS (35/77)	
Median value	6 (IQR: 4–7)
2–4 (mild)	12 (34.3%)
5–6 (moderate)	14 (40%)
≥7 (severe)	9 (25.7%)

Abbreviations: BUS, bowel ultrasound; CRP, C‐reactive protein; IBUS‐SAS: International Bowel Ultrasound Segmental Activity Score; IQR, interquartile range; MUC, Milan ultrasound criteria; PUCAI, pediatric ulcerative colitis activity index; TNF‐α, tumor necrosis factor alpha; UCEIS, ulcerative colitis endoscopic index of severity.

In terms of ultrasound findings, the most frequently observed feature was increased CWT (>3 mm), present in 96.1% of cases (74/77), with a median value of 5 mm (IQR: 4–6.5 mm), followed by enhanced vascularization, observed in 90.9% of cases (70/77). Conversely, loss of CWS was the least frequently observed finding (19.5%, 15/77). The median Limberg score was 3 (IQR 2.5–3), the median MUC was 8.3 (IQR 7.6–11.2), and the median IBUS‐SAS was 52 (IQR 34–70).

For 35 patients, a full colonoscopy was performed within 4 weeks before or after BUS, allowing direct comparison with endoscopy. Median time between BUS and endoscopy was 4 days (IQR − 1−8). The median Mayo endoscopic score was 2 (IQR 2–3), and the median UCEIS score was 6 (IQR 4–7).

**Figure 1:**
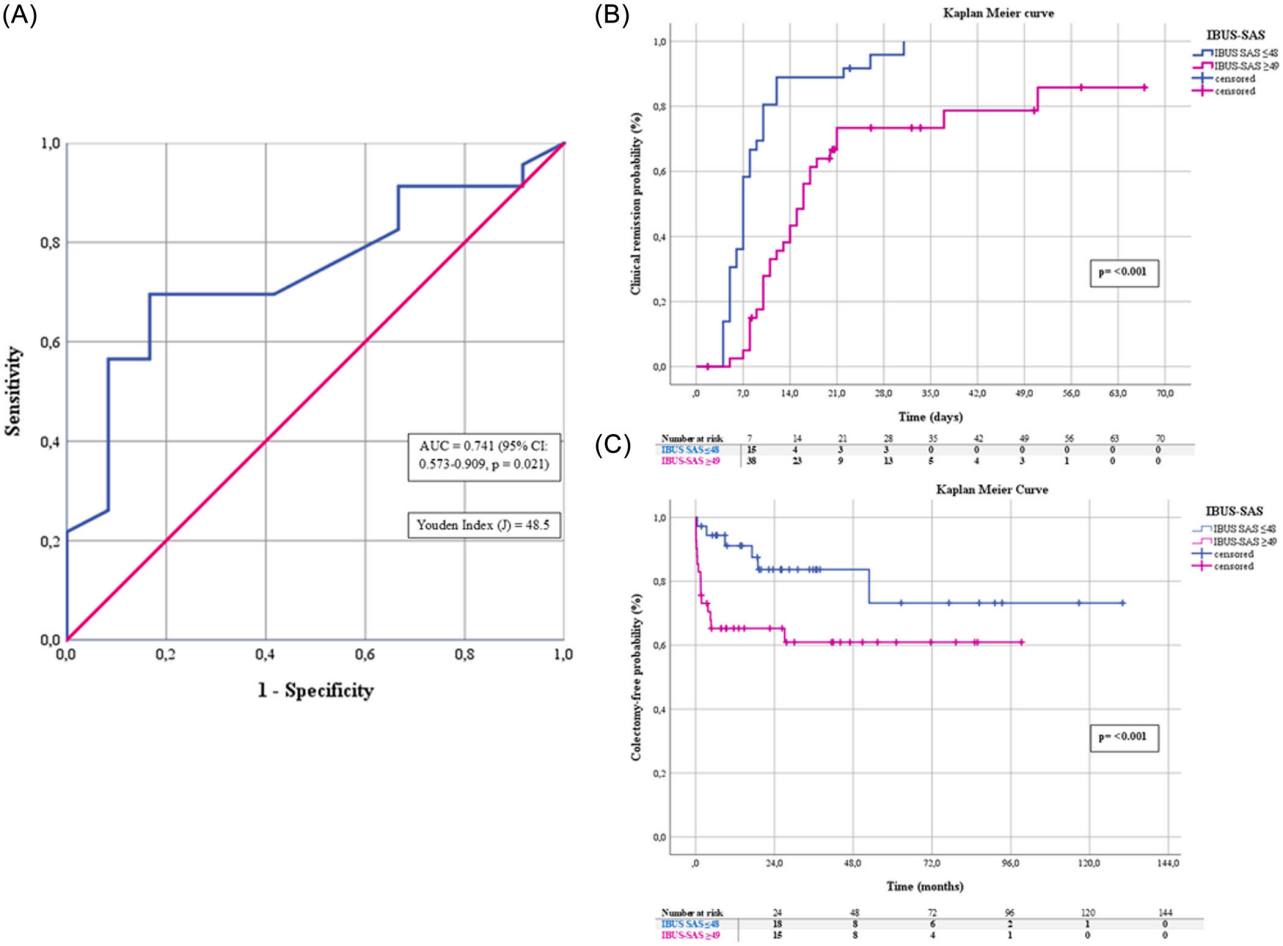
(A) ROC curves to detect moderate‐to‐severe endoscopic activity using IBUS‐SAS. (B) Kaplan–Meier curve of clinical remission probability based on IBUS‐SAS cut‐off. (C) Kaplan–Meier curve of colectomy‐free probability based on IBUS‐SAS cut‐off. IBUS‐SAS, International Bowel Ultrasound Segmental Activity Score; ROC, receiver operating characteristic.

A Spearman′s rank correlation analysis revealed a moderate positive correlation between IBUS‐SAS and UCEIS (*ρ* = 0.350, *p* = 0.039). The IBUS‐SAS demonstrated good ability to detect endoscopic activity of at least moderate severity (UCEIS ≥ 5) (Figure 1A). ROC analysis yielded an area under the curve (AUC) of 0.741 (95% CI: 73–0.909, *p* = 0.021). To further compare IBUS‐SAS with other ultrasound parameters and scores, ROC analyses were performed for CWT and the MUC, yielding AUCs of 0.632 (95% CI: 0.437–0.828, *p* = 0.205) for CWT and 0.605 (95% CI: 0.410–0.800, *p* = 0.099) for MUC.

The optimal IBUS‐SAS cut‐off for distinguishing moderate endoscopic activity was 48.5 (sensitivity 69.6%, specificity 83.3%) (Figure 1). Based on the cut‐off value of 48.5, the ultrasound cohort was divided into two groups (Table S1). The first group consisted of BUS with 0 IBUS‐SAS scores of 48 or below, while the second group included those with IBUS‐SAS values of 49 or higher. A comparative analysis between these two groups revealed no statistically significant differences in key clinical or biochemical parameters. Specifically, there were no significant variations in the median PUCAI or CRP levels at the time of the ultrasound examination. Additionally, no significant differences were observed in medical management strategies, including systemic steroid use (*p* = 0.977), initiation of step‐up therapy (*p* = 0.915), or hospitalization rates (*p* = 0.392). Moreover, when evaluating both short‐ and long‐term outcomes, significant differences were observed in terms of clinical remission rates and the need for colectomy within 12 months following BUS (*p* = 0.004 and *p* = 0.012, respectively).

Sixty‐five patients (84.4%) achieved clinical remission following moderate disease onset or flare. Kaplan–Meier survival analysis was performed (Figure 1B) revealed a significant difference in the probability of achieving clinical remission over time between the two groups (*p* < 0.001). A Cox regression analysis was conducted to identify independent predictors of clinical remission (Table 2). In the univariate analysis, only BUS parameters emerged as significant negative predictors of clinical remission. Specifically, the presence of CWT was associated with a HR of 0.17 (95% CI: 0.05–0.60, *p* = 0.005); CWT measured in millimeters showed an HR of 0.76 (95% CI: 0.65–0.90, *p* = 0.001); loss of CWS had an HR of 0.47 (95% CI: 0.24–0.94, *p* = 0.029); and the presence of i‐fat was associated with an HR of 0.57 (95% CI: 0.35–0.94, *p* = 0.029). IBUS‐SAS, Limberg, and MUC scores (as continuous variables) were also negative predictors of clinical remission (HR 0.97, 95% CI: 0.95–0.98, *p* < 0.001; HR 0.70, 95% CI: 0.54–0.89, *p* = 0.005; HR 0.86, 95% CI: 0.77–0.95, *p* = 0.003, respectively). IBUS‐SAS score ≥49 was with a lower likelihood of achieving clinical remission (HR: 0.31, 95% CI: 0.18–0.52, *p* < 0.001). Notably, none of the clinical parameters or medical treatment strategies evaluated were significant predictors of clinical remission. In the multivariate model—including PUCAI at the time of BUS, CWT (mm), loss of CWS, MUC, and IBUS‐SAS ≥ 49—only IBUS‐SAS ≥ 49 remained independent negative predictor of clinical remission (aHR: 0.42, 95% CI: 0.23–0.78, *p* = 0.003).

**Table 2:** Predictors of clinical remission in overall cohort.

	Clinical remission (*n* = 65, 84.4%)			
	Univariate analysis HR (95% CI)	*p*‐Value	Multivariate analysis HR (95% CI)	*p*‐Value
Time between diagnosis and BUS, months	1.00 (0.99–1.01)	0.861		
BUS indication (disease′s relapse)	1.12 (0.68–1.82)	0.649		
PUCAI at BUS time	0.97 (0.93–1.00)	0.089	0.99 (0.96–1.04)	0.861
CRP at BUS time, mg/dL	0.96 (0.88–1.05)	0.455		
Fecal calprotectin at BUS time, mg/kg	1.00 (1.00–1.00)	0.795		
BUS findings				
Thickened colonic wall	0.17 (0.05–0.60)	0.005	0.49 (0.23–1.03)	0.062
Colonic wall thickness, mm	0.76 (0.65–0.90)	0.001	0.72 (0.33–1.55)	0.507
Enhanced vascularization	0.86 (0.34–2.17)	0.763		
Colonic wall stratification loss	0.47 (0.24–0.92)	0.027		
Inflammatory mesenteric fat	0.57 (0.35–0.94)	0.029		
Enlarged mesenteric lymph nodes	0.79 (0.46–1.33)	0.379		
Limberg score	0.70 (0.54–0.89)	0.005		
MUC	0.86 (0.77–0.95)	0.003	0.88 (0.35–1.15)	0.181
IBUS‐SAS	0.97 (0.95–0.98)	<0.001		
IBUS‐SAS ≥ 49	0.31 (0.18–0.52)	<0.001	0.42 (0.23–0.78)	0.003
Therapeutic change after BUS				
Steroids	1.22 (0.75–1.99)	0.418		
Step‐up therapy	0.95 (0.54–1.67)	0.860		

Abbreviations: BUS, bowel ultrasound; CI, confidence interval; CRP, C‐reactive protein; HR, hazard ratio; IBUS‐SAS, international bowel ultrasound segmental activity score; MUC, Milan ultrasound criteria; PUCAI, pediatric ulcerative colitis activity index.

Seventeen of 77 patients (22%) required colectomy within 12 months after BUS. Notably, 12 of these 17 patients underwent surgery because they did not achieve clinical remission; the remaining five patients achieved clinical remission but experienced a subsequent relapse during the year following BUS, ultimately requiring colectomy due to multi‐refractory disease despite medical therapy. Kaplan–Meier analysis (Figure 1C) demonstrated a significant difference in colectomy‐free survival between the two groups over time (*p* = 0.031). Logistic regression analysis identified predictors of colectomy within 12 months following BUS (Table 3). In the univariate analysis, CWT (mm), Limberg score, IBUS‐SAS (as a continuous variable), and IBUS‐SAS ≥ 49 all emerged as significant predictors of colectomy. In the multivariate model, both CWT and IBUS‐SAS ≥ 49 remained independent predictors of colectomy within 12 months after BUS. Specifically, CWT was associated with an OR of 1.50 (95% CI: 1.04–2.18, *p* = 0.029), and an IBUS‐SAS score ≥49 was associated with an OR of 4.18 (95% CI: 1.03–16.91, *p* = 0.045).

**Table 3:** Predictors of colectomy within 12 months of follow‐up in overall cohort.

	Colectomy at 1 year (*n* = 17, 22%)			
	Univariate analysis OR (95% CI)	*p*‐Value	Multivariate analysis OR (95% CI)	*p*‐Value
Time between diagnosis and BUS, months	1.00 (0.97–1.02)	0.895		
BUS indication (disease′s relapse)	0.58 (0.19–1.78)	0.344		
PUCAI at BUS time	1.07 (0.99–1.15)	0.082		
CRP at BUS time, mg/dL	1.10 (0.93–1.30)	0.246		
Fecal calprotectin at BUS time, mg/kg	1.001 (1.00–1.01)	0.231		
BUS findings				
Thickened colonic wall	0 (0–0)	0.999	1.5 (1.04–2.18)	0.029
Colonic wall thickness, mm	1.63 (1.14–2.32)	0.006		
Enhanced vascularization	1.46 (0.25–8.32)	0.666		
Colonic wall stratification loss	2.08 (0.60–7.23)	0.248		
Inflammatory mesenteric fat	2.4 (0.75–7.65)	0.139		
Enlarged mesenteric lymph nodes	0.66 (0.21–2.00)	0.466		
Limberg score	4.77 (1.56–14.5)	0.006		
MUC	1.15 (0.94–1.41)	0.169		
IBUS‐SAS	1.4 (1.01–1.8)	0.003		
IBUS‐SAS ≥ 49	5.7 (1.48–21.92)	0.011	4.18 (1.03–16.91)	0.045
Therapeutic change after BUS				
Steroids	2.5 (0.51–12.2)	0.258		
Step‐up therapy	0.34 (0.10–1.08)	0.069		

Abbreviations: BUS, bowel ultrasound; CI, confidence interval; CRP, C‐reactive protein; HR, hazard ratio; IBUS‐SAS, international bowel ultrasound segmental activity score; MUC, Milan ultrasound criteria; OR, odds ratio; PUCAI, pediatric ulcerative colitis activity index.

## 4 DISCUSSION

This study demonstrates that BUS may represent an effective and non‐invasive tool for improving risk stratification in pediatric UC, particularly in moderate disease activity, with implications for both short‐ and long‐term outcomes. In this setting, the IBUS‐SAS proved applicable also in pediatric UC, allowing the identification of high‐risk patients within the intermediate range of clinical activity (PUCAI 35–60), a subgroup currently managed uniformly according to guidelines.^1^

In recent years, evidence supports the role of BUS in evaluating inflammatory activity in UC. The 2025 ESPGHAN guidelines emphasize the potential use of ultrasound in monitoring patients with UC, particularly for the management of ASC.^1^, ^2^, ^15^ In this context, our group first demonstrated in a retrospective study^15^ and later confirmed in a prospective study,^24^ that BUS is feasible in ASC, predicting first‐line therapy failure and the need for colectomy, outperforming PUCAI. Conversely, data in mild‐to‐moderate pediatric UC remain limited. In our cohort, CWT and increased vascularity were observed in nearly all patients. In contrast, loss of CWT, considered a more specific marker of severe and/or chronic inflammation, was identified in approximately 20% of patients. This supports the ability of BUS to detect inflammation even in moderate disease (and not only severe).

To further investigate the potential of BUS in stratifying this heterogeneous group, we evaluated the performance of the IBUS‐SAS, recently evaluated in adults with UC. This score incorporates four key ultrasound parameters and includes a scoring system ranging from 0 to 100, making it more granular than other UC‐specific ultrasound scores such as the MUC,^17^ the UC‐IUS index,^18^ and the Civitelli index.^20^ Our findings showed a moderate correlation between IBUS‐SAS and endoscopic activity, particularly in patients with UCEIS ≥ 5, with an AUC of 0.741 (*p* = 0.021) and an optimal cut‐off of 48.5 (sensitivity 69.6%, specificity 83.3%). In adults, Fischer et al.^23^ reported high diagnostic accuracy for identifying moderate disease activity with an IBUS‐SAS cut‐off of 38.4 (AUC 0.83; sensitivity 78.3%, specificity 81.8%). Similarly, Innocenti et al.^30^ found a strong correlation between IBUS‐SAS and both MES (*ρ* = 0.72) and UCEIS (*ρ* = 0.73), with AUCs of 0.87 and 0.89, respectively, for detecting at least moderate endoscopic activity, using thresholds of >19 and >23. Notably, both of these studies based the IBUS‐SAS calculation on the sigmoid colon. In contrast, we used the segment with the worst ultrasound findings, which may better reflect the typical pancolonic distribution of pediatric UC. In children, IBUS‐SAS was recently used for the first time in a study by Dolinger et al.,^31^ which primarily aimed to assess early ultrasound changes following initiation of biologics or small molecules. In that study, an IBUS‐SAS value ≤8.4 at 6‐month follow‐up demonstrated excellent diagnostic accuracy for identifying endoscopic remission. In addition, we compared the diagnostic performance of IBUS‐SAS with other ultrasound scores and individual parameters, with ROC analyses demonstrating that IBUS‐SAS outperformed both the MUC score and CWT alone, with AUCs of 0.741 (*p* = 0.021) versus 0.605 (*p* = 0.099) and 0.632 (*p* = 0.205), respectively. Several factors may account for these findings, particularly considering that the MUC has been reported to correlate strongly with endoscopic activity.^17^, ^30^ First, the ROC analysis was conducted in a relatively small sample. Second, our cohort was highly selected, including only patients with clinically moderate disease and a restricted distribution of endoscopic severity. MUC values were consequently clustered (median 8.3, IQR: 7.6–11.2), with most patients exhibiting vascular signals, so that CWT accounted for most of the score variability. This limited dispersion, likely reflecting the clinical profile of our cohort, may have reduced the discriminative performance of the MUC. In contrast, the wider scoring range and greater granularity of the IBUS‐SAS may have allowed it to better capture subtle differences, despite the small sample size.

After identifying the cut‐off, we applied this threshold to the overall cohort. Importantly, although patients with IBUS‐SAS ≥ 49 did not differ from those below the cut‐off in baseline PUCAI scores or CRP levels, they experienced significantly lower rates of clinical remission (*p* = 0.004) and a higher risk of colectomy within 12 months (*p* = 0.012). These differences were clearly illustrated by the Kaplan–Meier survival curves, which demonstrated a significant divergence between the two groups for these two outcomes (*p* = 0.001 and *p* = 0.031, respectively). Notably, only ultrasound‐derived parameters emerged as statistically significant predictors in both contexts. In the multivariate Cox regression model, which adjusted for PUCAI at the time of BUS, IBUS‐SAS ≥ 49 (HR: 0.42, *p* = 0.003) remained independent predictor of a lower likelihood of achieving clinical remission. Similarly, in the multivariate logistic regression model, both CWT (OR: 1.50, *p* = 0.029) and IBUS‐SAS ≥ 49 (OR: 4.18, *p* = 0.045) remained independent predictors of colectomy within the first year after BUS. These findings underscore the potential value of this scoring system in improving the stratification of patients who may appear clinically and laboratoristically homogeneous, but ultimately experience markedly different short‐ and long‐term outcomes.

The present study has several limitations. First, its retrospective design introduces potential selection and information bias, as patients with more severe disease (median PUCAI 45) or refractory disease (many receiving first‐ or second‐line biological therapy or with prior severe flares) may have been preferentially referred for BUS, potentially contributing to the relatively high colectomy rate and limiting generalizability. Second, although BUS at our institution is performed using a structured reporting system, all scores evaluated (IBUS‐SAS and MUC) were calculated retrospectively, whereas the Civitelli Index and UC‐IUS—potentially useful for comparison—could not be reliably assessed because both require evaluation of colonic haustrations, not routinely documented in the ultrasound reports. Third, endoscopic evaluations were not performed in all patients, which could have affected the accuracy of reported associations and the proposed IBUS‐SAS cut‐off, limiting generalizability. Finally, although the median interval between ultrasound and endoscopy was very short (4 days), potential influences from recently initiated treatments cannot be entirely excluded; to mitigate this, patients who had started or modified immunomodulators, corticosteroids, or biologics within predefined timeframes were excluded.

## 5 CONCLUSION

BUS is a promising non‐invasive tool for monitoring disease activity in pediatric UC. The IBUS‐SAS demonstrated good correlation with endoscopic severity and meaningful prognostic value, particularly in identifying high‐risk patients within clinically intermediate disease categories.

However, further prospective validation in larger and more heterogeneous pediatric cohorts is warranted to confirm these findings and support the integration of BUS and IBUS‐SAS into routine clinical practice, with the goal of optimizing medical management and improving long‐term outcomes.

## CONFLICT OF INTEREST STATEMENT

The authors declare no conflicts of interest.

## Supporting information

Supplemental Table 1. Comparison of clinical characteristics based on IBUS‐SAS cut‐off.
