# Supplementary material for: Bowel ultrasound scan in the management of pediatric moderate ulcerative colitis: The applicability of international bowel ultrasound segmental activity score
Source: J Pediatr Gastroenterol Nutr. 2026 Jun 8;83(3):438–46. doi: 10.1002/jpn3.70476 (PMC13539686; doi:10.1002/jpn3.70476)
Supplement: Supplementary file 1 [file JPN3-83-438-s001.docx]

**Supplemental Table 1.** **Comparison of clinical characteristics based on IBUS-SAS cut-off.**

|  | Overall cohort (n=77) | |  |
| --- | --- | --- | --- |
|  | **IBUS SAS ≤48**  **(n=36; 46.7%)** | **IBUS-SAS ≥49**  **(n=41; 53.3%)** | **p-value** |
| Median PUCAI at BUS moment | 45 (IQR:40-50) | 50 (IQR:45-55) | 0.079 |
| Median CRP at BUS moment (mg/dl) | 1 (IQR:0-1.7) | 1.1 (0,2-3.6) | 0.056 |
| Medication at BUS time  No medication  Steroids  Mesalamine  Azathioprine  Infliximab  Adalimumab  Vedolizumab  Ustekinumab | 14 (38.9%)  14 (38.9%)  21 (58.3%)  6 (16.7%)  8 (22.2%)  2 (5.6%)  2 (5.6%)  0 (0%) | 11 (26.8%)  21 (51.2%)  26 (63.4%)  9 (22%)  14 (34.1%)  1 (2.4%)  5 (12.2%)  1 (2.4%) | 0.259  0.278  0.648  0.559  0.248  0.596  0.438  1 |
| BUS indication  Disease’s onset  Disease’s relapse | 13 (36.1%)  23 (63.9%) | 22 (53.7%)  19 (46.3%) | 0.123 |
| Median time between diagnosis and BUS (months) | 2.2 (0.1-12.5) | 3.7 (0.1-16) | 0.540 |
| Median UCEIS (35/77) | 5 (IQR:4-6) | 6 (IQR:5-7) | **0.049** |
| Median MES (35/77) | 2 (IQR:1-2) | 3 (IQR:2-3) | **0.014** |
| Therapeutic change |  |  |  |
| Steroids  Step-up therapy | 28 (77.8%)  18 (50%) | 32 (78%)  20 (48.8%) | 0.977  0.915 |
| Hospitalization | 27 (75%) | 34 (82.9%) | 0.392 |
| Clinical remission | 35 (97.2%) | 30 (73.2%) | **0.004** |
| Colectomy within 12 months | 3 (8.3%) | 14 (34.1%) | **0.012** |

**BUS:** Bowel Ultrasound. **CRP:** C reactive protein. **IBUS-SAS:** International Bowel Ultrasound Segmental Activity Score. **MES:** Mayo Endoscopic Score. **PUCAI:** Pediatric Ulcerative Colitis Activity Index. **UCEIS:** Ulcerative Colitis Endoscopic Index of Severity.
